# Supplementary material for: TAGET: a toolkit for analyzing full-length transcripts from long-read sequencing
Source: Nat Commun. 2023 Sep 23;14:5935. doi: 10.1038/s41467-023-41649-0 (PMC10518008; doi:10.1038/s41467-023-41649-0)
Supplement: Supplementary file 1 — Supplementary Information [file 41467_2023_41649_MOESM1_ESM.pdf]

Supplementary Information for
TAGET: A toolkit for analyzing full-length transcripts from long-read sequencing

Yuchao Xia[1,2,#], Zijie Jin[3,4,#], Chengsheng Zhang[2,#], Linkun Ouyang[5,#], Yuhao Dong[2], Juan Li[6], Lvze Guo[2], Biyang Jing[2], Yang Shi[7], Susheng Miao[8,*], Ruibin Xi[4,5,9,*]

[1]College of Science, Beijing Information Science and Technology University, Beijing, 100192, China

[2]Beijing GeneX Health Co.,Ltd, Beijing, 100195, China

[3]Peking University International Cancer Institute, Health Science Center, Peking University, Beijing, 100191, China

[4]School of Mathematical Sciences, Peking University, Beijing, 100871, China

[5]Academy for Advanced Interdisciplinary Studies, Peking University, Beijing, 100871, China

[6]Department of Biomedical Engineering, College of Future Technology, Peking University, Beijing, 100871, China

[7]BeiGene (Beijing) Co., Ltd., Beijing, China

[8]Department of Head and Neck Surgery, Harbin Medical University Cancer Hospital, Harbin, 150081, China

[9]Center for Statistical Science, Peking University, Beijing, 100871, China

*To whom correspondence should be addressed. Email: drmiaosusheng@126.com and ruibinxi@math.pku.edu.cn

[#]These authors contributed equally.

This supplementary file includes:

Supplementary Figure 1 to 12

Other supplementary materials for this manuscript include the following:

Supplementary Table 1 to 5

Supplementary Figures

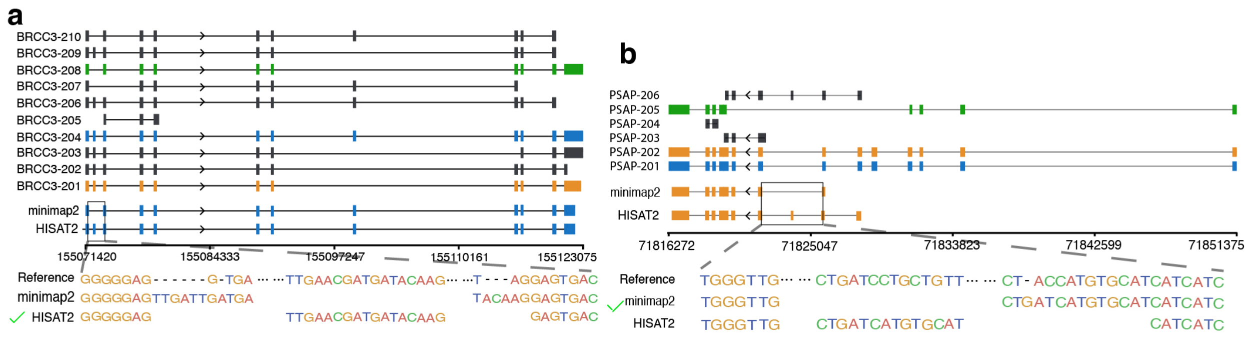

Supplementary Figure 1. Examples of incorrect mapping given by long-read and short-read alignment methods. (a) minimap2 gives a more suitable mapping than HISAT2. **(b)** HISAT2 gives a more suitable mapping than minimap2.

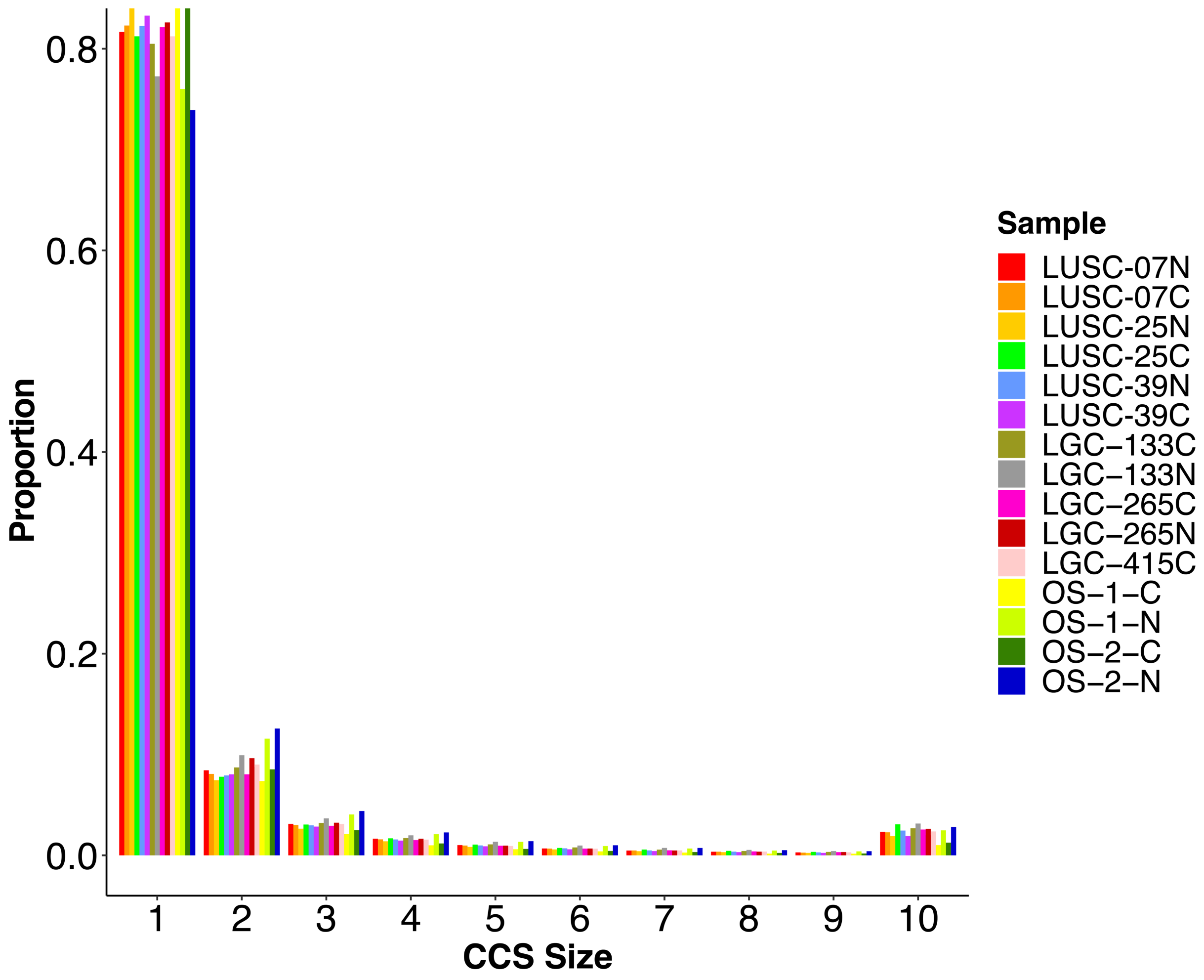

Supplementary Figure 2. The CCS size distribution of fifteen samples.

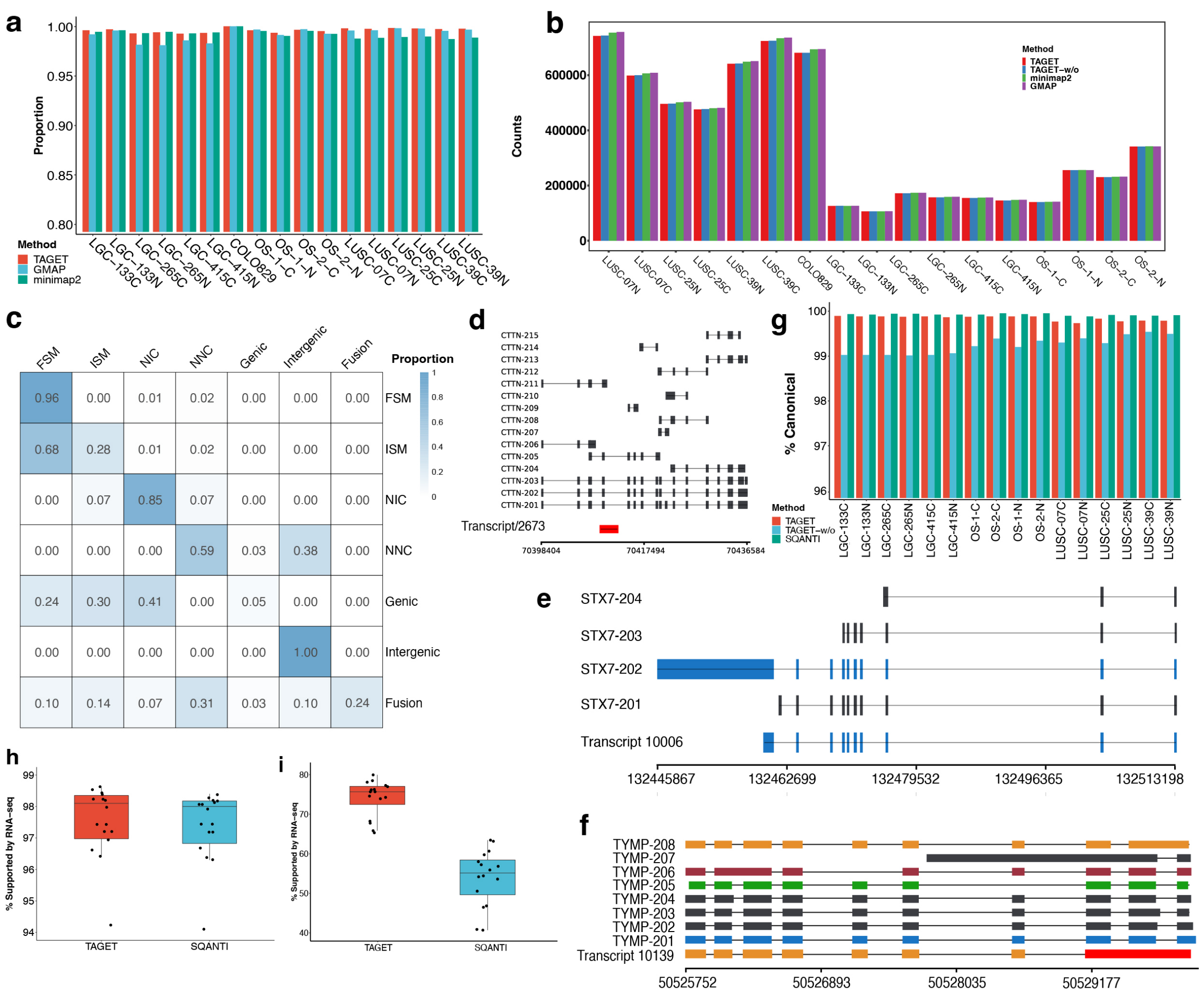

Supplementary Figure 3. Performance comparison on mapping and annotation. (a) The proportions of reads that are mapped to the reference genome by TAGET, GMAP, and minimap2 (CCS reads in clusters of size ⩾ 2). **(b)** The numbers of splice junctions reported by TAGET, TAGET-w/o, GMAP, and minimap2 (junctions supported by multiple reads were counted multiple times). **(c)** Consistent heatmap of SQANTI and TAGET annotations. Rows and columns are the classifications by TAGET and SQANTI, respectively. Numbers in the heatmap represent proportions of a TAGET classification being classified as a transcript class by SQANTI. For example, 0.96 at the top left panel means that 96% of the TAGET FSMs are also classified as FSM by SQANTI. **(d)** A monoexonic transcript 2673 was annotated as Genic by TAGET and as NIC by SQANTI. **(e)** The transcript 10006 was annotated as FSM by SQANTI and ISM by TAGET. Transcript 10006 matches every splice junction of the reference transcript STX7-202, but it does not fully cover the first exon of STX7-202. **(f)** Transcript 10139 was annotated as FSM by SQANTI and NIC by TAGET. Transcript 10139 is most similar to TYMP-208, but compared to TYMP-208,

transcript 10139 has an intron retention. **(g)** Proportions of canonical junctions in junctions reported by TAGET, TAGET-w/o, and SQANTI. Each point represents a dataset. **(h)** The percentage of all splice junctions supported by at least two short reads (n=16 samples). **(i)** The percentage of novel splice junctions supported by at least two short reads (n=16 samples). The center line in the boxplot is the median, bounds of boxes are the interquartile of the data, whiskers represent minima/maxima excluding outliers and dots represents outliers of beyond 1.5*Interquartile Range (IQR) from either end of the box.

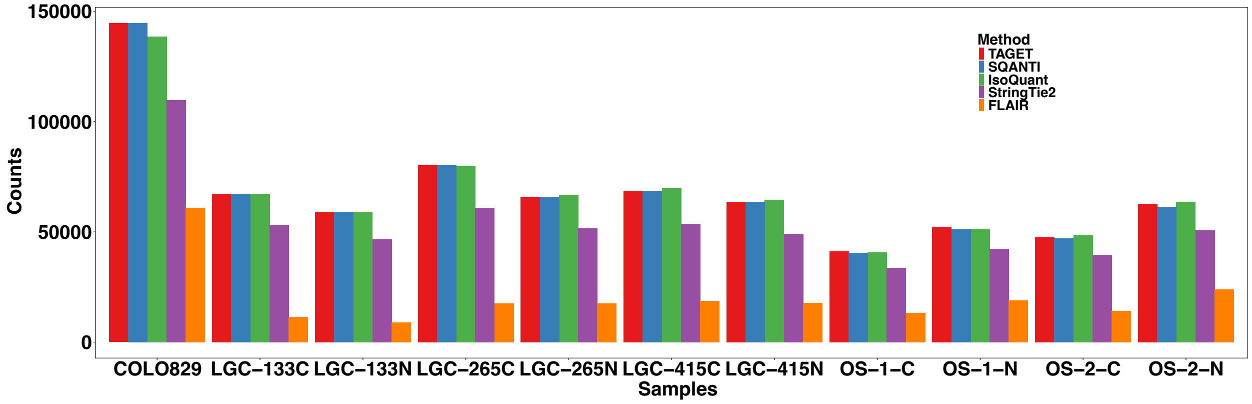

Supplementary Figure 4. The number of unique junctions reported by TAGET and SQANTI.

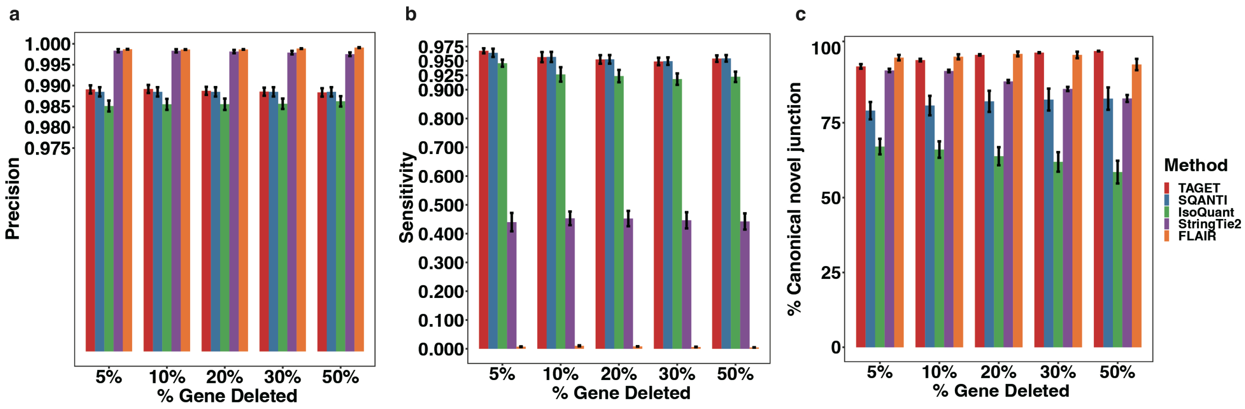

Supplementary Figure 5. The sensitivity and precision of TAGET, SQANTI, IsoQuant, StringTie2, and FLAIR using incomplete annotations (n=11 samples). (a) Mean sensitivities of different algorithms using annotations with different percentages of genes deleted. **(b)** Mean precisions of different algorithms using different incomplete annotations. **(c)** The proportions of canonical junctions in novel junctions using different incomplete annotations. Error bars represent the standard deviation of independent samples.

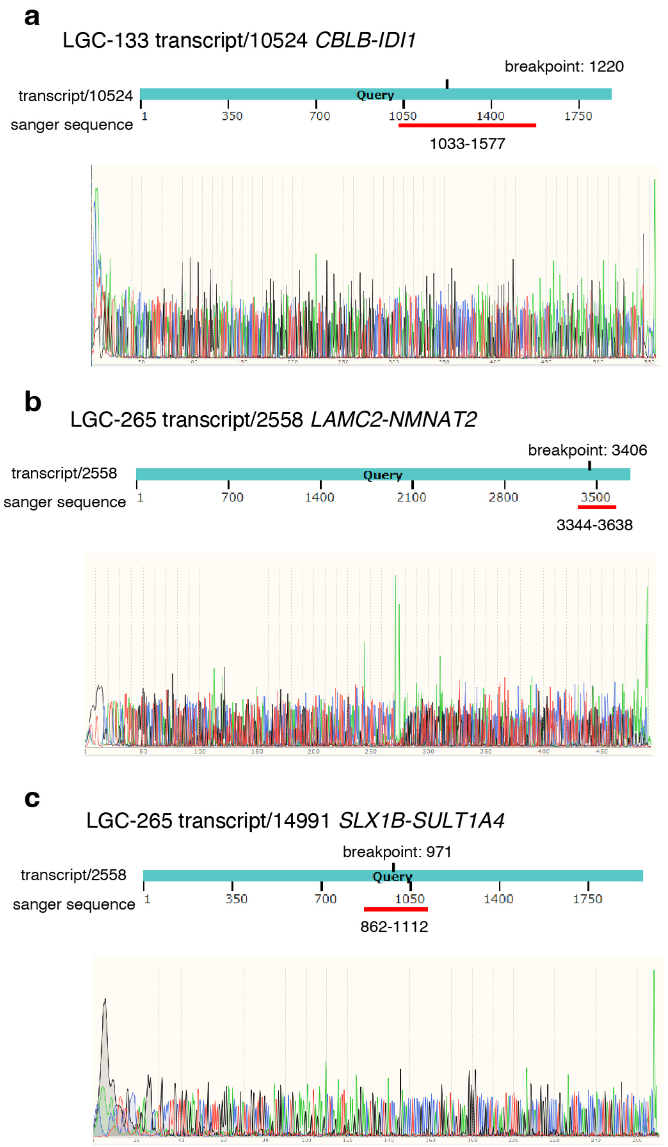

Supplementary Figure 6. Validation of the fusions by Sanger Sequencing. (a) The validation of *CBLB-IDI1* gene fusion; **(b)** The validation of *LAMC2-NMNAT2* gene fusion; **(c)** The validation of *SLX1B-SULT1A4* gene fusion.

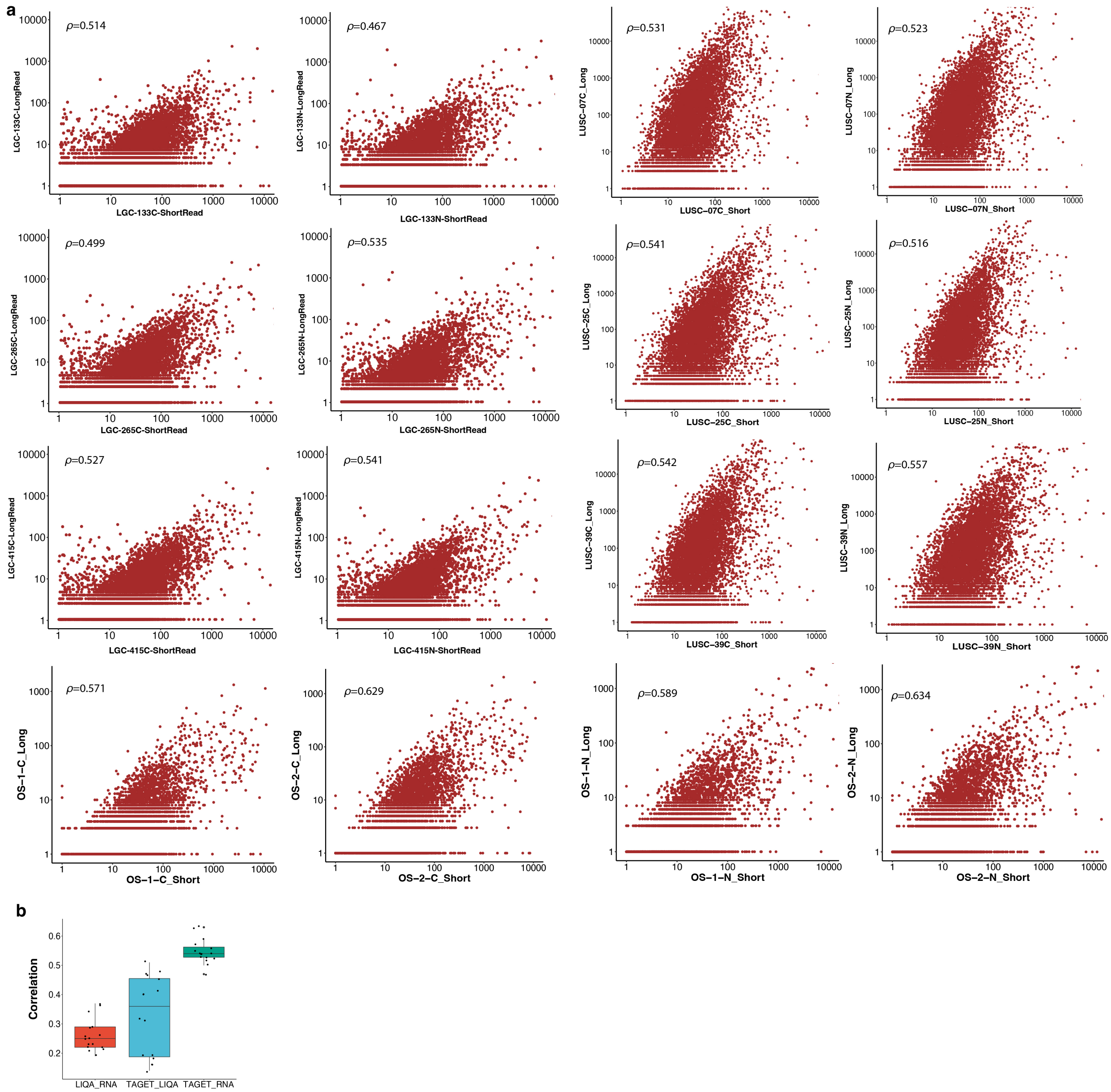

Supplementary Figure 7. Correlations between Iso-seq and RNA-seq expressions. (a) The scatterplots of Iso-seq expression against RNA-seq expression. **(b)** The Spearman's correlation of gene expression from Iso-seq data and RNA-seq data given by TAGET and LIQA, respectively (n=16 samples). The center line in the boxplot is the median, bounds of boxes are the interquartile of the data, whiskers represent minima/maxima excluding outliers and dots represents outliers of beyond 1.5*Interquartile Range (IQR) from either end of the box.

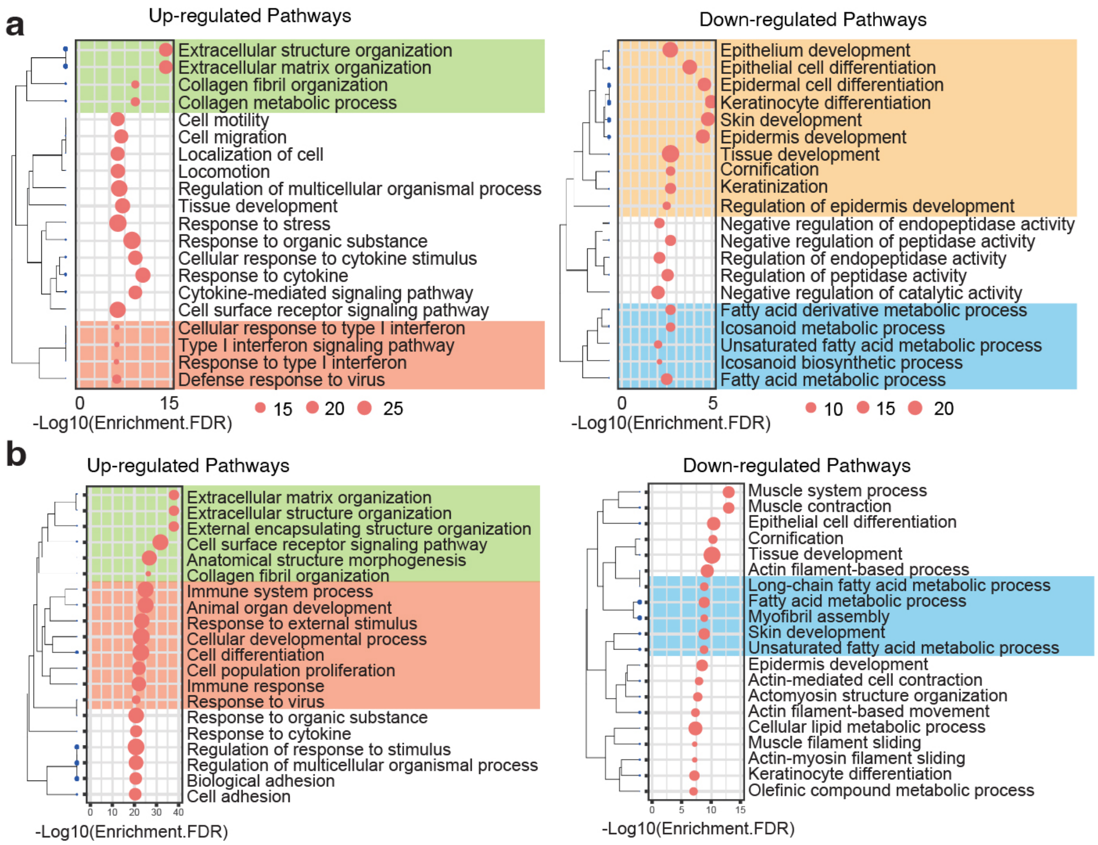

Supplementary Figure 8. The Gene ontology (GO) enrichment analysis of up- and down-regulated genes (FDR < 0.05) between three pairs of LGC samples. (a) The GO enrichment analysis using Iso-seq data **(b)** The GO enrichment analysis using RNA-seq data. The sizes of dots indicate the number of genes in the enriched pathway.

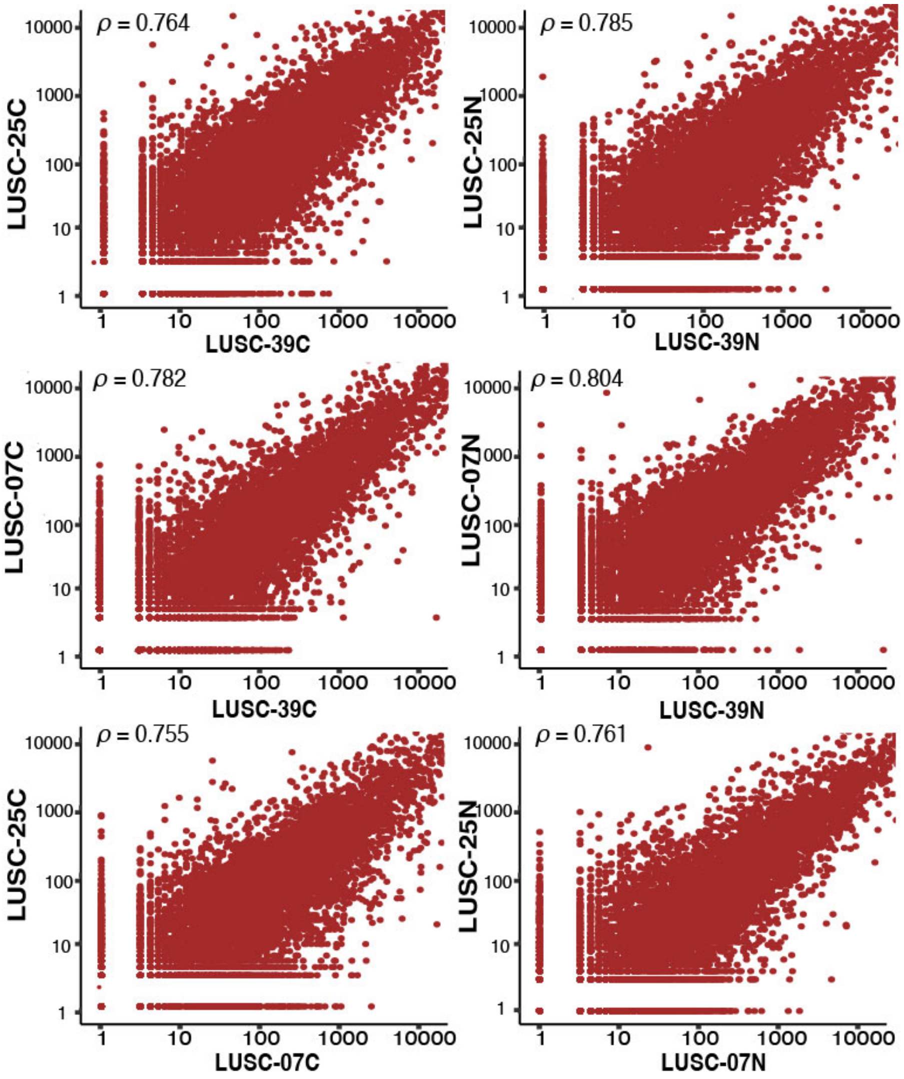

Supplementary Figure 9. Scatter plots of Iso-seq expressions between pairs of lung squamous cell carcinoma normal/tumor samples.

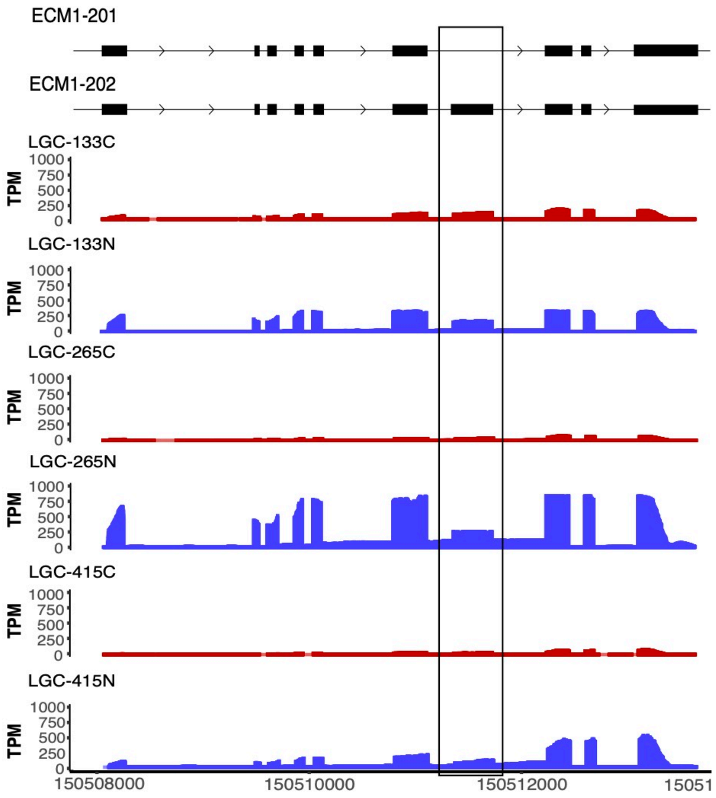

Supplementary Figure 10. Expression of ECM1 from paired cancer and normal samples in RNA-seq data. The black box indicates the region that two isoforms are different.

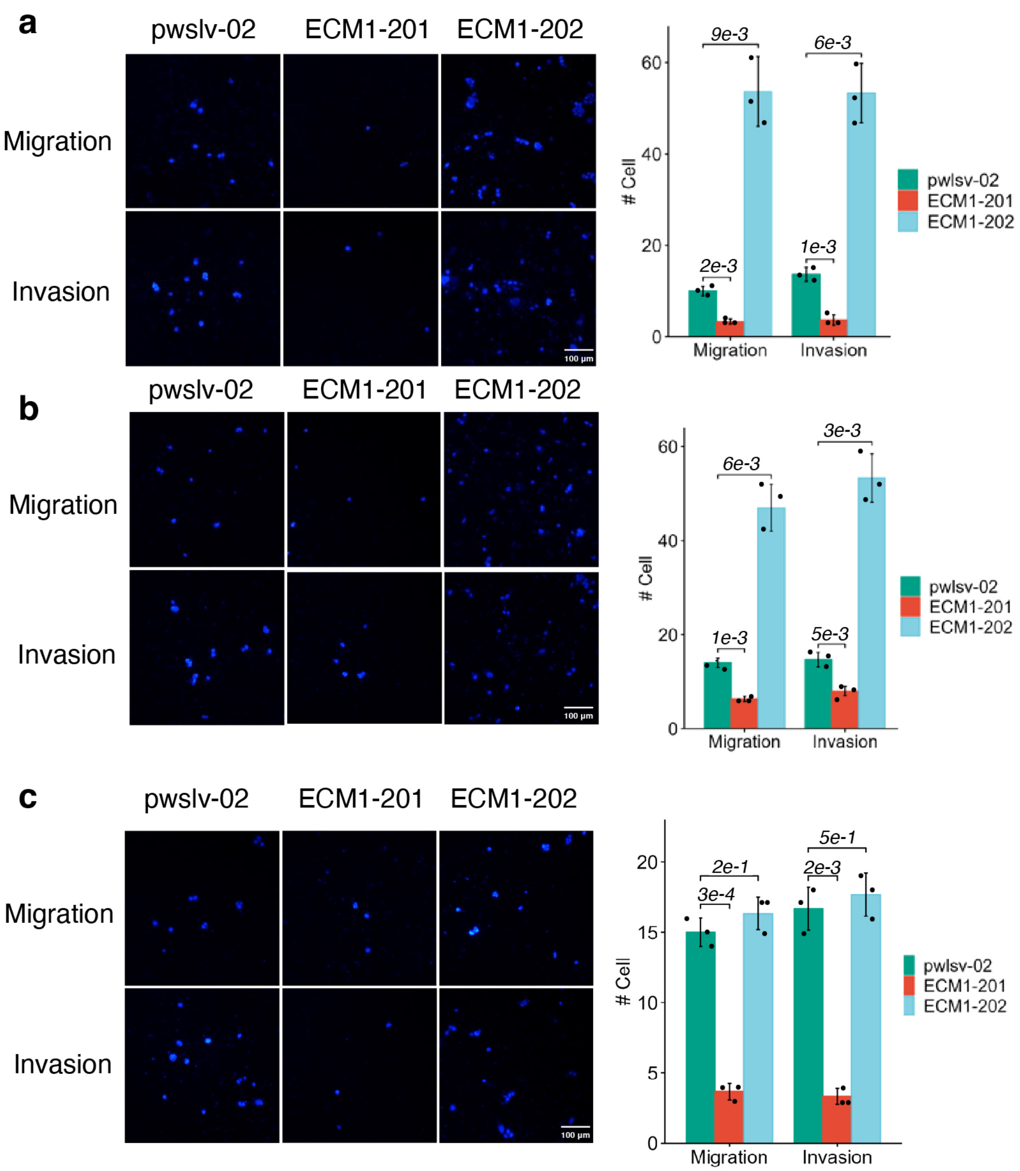

Supplementary Figure 11. Effects of ECM1-201 and ECM1-202 isoforms on cell migration and invasion using the Transwell system. Three cell lines were tested: HeyA8 **(a)**, AU565 **(b)**, and 5-8F **(c).** Error bars represent the standard deviation of three independent experiments (n=3 experiments; two-sided Student's t-test; **: $p < 0.01$; ***: $p < 0.001$).

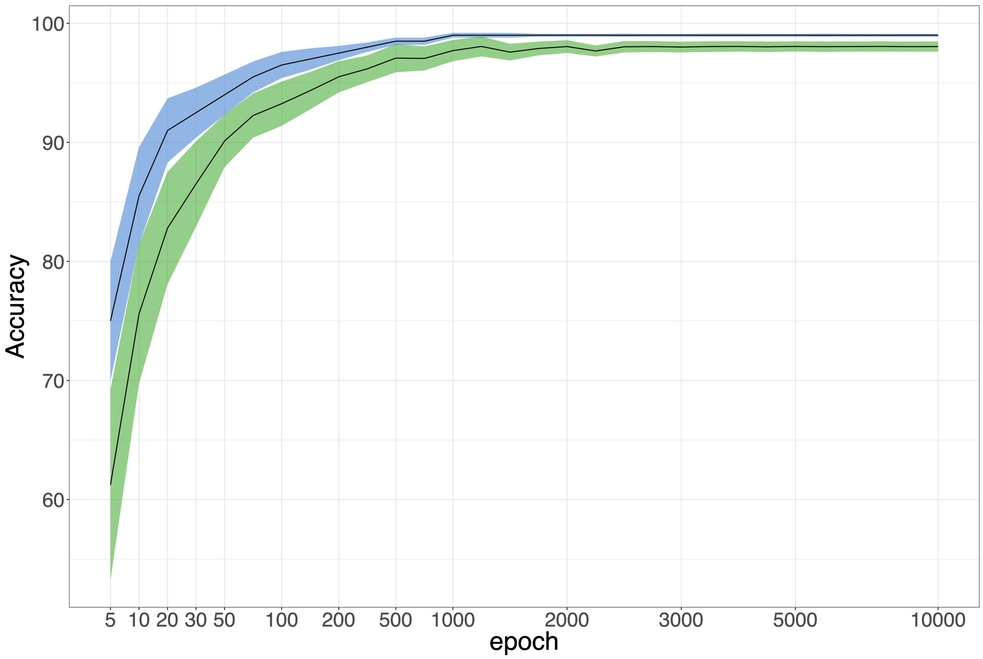

Supplementary Figure 12. Accuracy plot of the CNN model in the training (blue) and testing (green) datasets.

Supplementary Tables

Supplementary Table 1. Total number of transcripts and sequencing depth in each sample.

Sample	#Transcript	Raw data size
LGC-133-C	16918	15G
LGC-133-N	14710	15G
LGC-415-C	28422	25G
LGC-415-N	28799	25G
LGC-265-C	26745	25G
LGC-265-N	27622	25G
OS-1-C	21113	35G
OS-1-N	31313	40G
OS-2-C	35060	40G
OS-2-N	40531	40G
COLO829	68553	NA
LUSC-07C	155225	120G
LUSC-07N	170331	120G
LUSC-25C	90176	120G
LUSC-25N	123249	120G
LUSC-39C	170611	120G
LUSC-39N	128619	120G

Supplementary Table 2. The number of all splice junctions supported by at least two short reads.

Sample	Method	Proportion	Number
LGC-133C	TAGET	0.98	123566
LGC-133C	SQANTI	0.979	123655
LGC-133N	TAGET	0.982	104358
LGC-133N	SQANTI	0.981	104607
LGC-265C	TAGET	0.964	165715
LGC-265C	SQANTI	0.963	167110
LGC-265N	TAGET	0.966	151894
LGC-265N	SQANTI	0.964	153120
LGC-415C	TAGET	0.969	150195
LGC-415C	SQANTI	0.967	151088
LGC-415N	TAGET	0.942	137445
LGC-415N	SQANTI	0.941	139150
OS-1-C	TAGET	0.972	136317
OS-1-C	SQANTI	0.972	136867
OS-2-C	TAGET	0.972	223332
OS-2-C	SQANTI	0.972	225103
OS-1-N	TAGET	0.974	248951
OS-1-N	SQANTI	0.974	249775
OS-2-N	TAGET	0.974	332534
OS-2-N	SQANTI	0.974	333605
LUSC-07C	TAGET	0.984	588664
LUSC-07C	SQANTI	0.982	595396
LUSC-07N	TAGET	0.982	728672
LUSC-07N	SQANTI	0.981	739348
LUSC-25C	TAGET	0.986	468800
LUSC-25C	SQANTI	0.984	472528
LUSC-25N	TAGET	0.984	487840
LUSC-25N	SQANTI	0.982	492340
LUSC-39C	TAGET	0.985	712593
LUSC-39C	SQANTI	0.983	721140
LUSC-39N	TAGET	0.982	629601
LUSC-39N	SQANTI	0.981	636156

Supplementary Table 3. The number of novel splice junctions supported by at least two short reads.

Sample	Method	Proportion	Number
LGC-133C	TAGET	0.773	2093
LGC-133C	SQANTI	0.631	1576
LGC-133N	TAGET	0.8	1893
LGC-133N	SQANTI	0.634	1323
LGC-265C	TAGET	0.739	6508
LGC-265C	SQANTI	0.606	4482
LGC-265N	TAGET	0.781	4260
LGC-265N	SQANTI	0.558	2265
LGC-415C	TAGET	0.757	3649
LGC-415C	SQANTI	0.535	1980
LGC-415N	TAGET	0.757	4341
LGC-415N	SQANTI	0.504	1987
OS-1-C	TAGET	0.682	1853
OS-1-C	SQANTI	0.407	825
OS-2-C	TAGET	0.658	3357
OS-2-C	SQANTI	0.467	1733
OS-1-N	TAGET	0.677	4608
OS-1-N	SQANTI	0.405	2398
OS-2-N	TAGET	0.653	5763
OS-2-N	SQANTI	0.463	3560
LUSC-07C	TAGET	0.762	20742
LUSC-07C	SQANTI	0.541	10649
LUSC-07N	TAGET	0.785	29142
LUSC-07N	SQANTI	0.597	15858
LUSC-25C	TAGET	0.743	12974
LUSC-25C	SQANTI	0.579	7757
LUSC-25N	TAGET	0.77	15290
LUSC-25N	SQANTI	0.567	8289
LUSC-39C	TAGET	0.746	24028
LUSC-39C	SQANTI	0.543	12277
LUSC-39N	TAGET	0.763	20446
LUSC-39N	SQANTI	0.571	11395

Supplementary Table 4. Pearson's correlations of gene expression between LIQA and TAGET.

Sample	Correlation
GM12878_1	0.96
GM12878_2	0.95
GM12878_3	0.95
Lung_1	0.9
Lung_2	0.77
Lung_3	0.87
Lung_4	0.92
Lung_5	0.81
Lung_6	0.8
Lung_7	0.76
Lung_8	0.87
Lung_9	0.92
Lung_10	0.58
Lung_11	0.89
Lung_12	0.61
Lung_13	0.69
Lung_14	0.73
Lung_15	0.77
Lung_16	0.76
Lung_17	0.93
Lung_18	0.64
Lung_19	0.89
Lung_20	0.78
Lung_21	0.75
Lung_22	0.82
Lung_23	0.79

Supplementary Table 5. The primer sequences of RT-qPCR for ECM1b (ECM1-201) and ECM1a (ECM1-202).

Index	Primer Name	Sequence (5' to 3')	# Bases
1	ECM1-F-202	GACAGAGTCAAGTGCAGCCC	20
2	ECM1-R-202	TCACAGAATCGGCTCATT	18
3	ECM1-F-201	AAACTTGTGTGGGAGGATAC	20
4	ECM1-R-201	GTCATAGTTGGGGTAAGGAG	20
