# Supplementary material for: TAGET: a toolkit for analyzing full-length transcripts from long-read sequencing
Source: Nat Commun. 2023 Sep 23;14:5935. doi: 10.1038/s41467-023-41649-0 (PMC10518008; doi:10.1038/s41467-023-41649-0)
Supplement: Supplementary file 3 — Description of Additional Supplementary Files [file 41467_2023_41649_MOESM3_ESM.pdf]

Description of Additional Supplementary Files

File Name: **Supplementary Data 1.**
Description: The number of reads that are mapped to the reference genome by different methods.

File Name: **Supplementary Data 2.**
Description: The number of splice sites bias reported by different methods.

File Name: **Supplementary Data 3.**
Description: The number of junctions validated by RNA-seq.

File Name: **Supplementary Data 4.**
Description: The number of canonical junctions reported by TAGET, TAGET-w/o, and SQANTI.

File Name: **Supplementary Data 5.**
Description: The number of canonical novel junction.

File Name: **Supplementary Data 6.**
Description: The validated novel isoforms of COLO829 and LGC-133C and their PCR primer sequences.

File Name: **Supplementary Data 7-10.**
Description: The bed files of transcripts involved in the experimental validation. Each line in the bed files means the alignment with a fragment of the transcript on genome. The Chr, Genome_start, and Genome_end columns represent the genome coordinates of the alignment results. The Transcript column labels the ID of each transcript, and the Strand column indicates the strand direction of genome alignment. Transcript_start_site and Transcript_end_site columns show the location of the partial transcript fragment. **Supplementary Data 7:** novel transcripts from COLO829 sample; **Supplementary Data 8:** novel transcripts from LGC-133C sample; **Supplementary Data 9:** novel transcripts from LUSC-39C sample; **Supplementary Data 10:** novel transcripts from LUSC-07C sample;

File Name: **Supplementary Data 11.**
Description: The validated somatic gene fusion of LGC-133 and LGC-265 and their PCR primer sequences.

File Name: **Supplementary Data 12-14.**
Description: The bed files of transcripts involved in the experimental validation. Each line in the bed files means the alignment with a fragment of the transcript on genome. The Chr, Genome_start, and Genome_end columns represent the genome coordinates of the alignment results. The Transcript column labels the ID of each transcript, and the Strand column indicates the strand direction of genome alignment. Transcript_start_site and Transcript_end_site columns show the location of the partial transcript fragment. **Supplementary Data 12:** somatic fusion transcripts from LGC-133C sample; **Supplementary Data 13:** somatic fusion transcripts from LGC-265C sample; **Supplementary Data 14:** somatic fusion transcripts from LUSC-25C sample.

File Name: **Supplementary Data 15.**
Description: The TPT value of isoforms in three pairs of laryngocarcinoma samples.

File Name: **Supplementary Data 16.**
Description: The TPT value of isoforms in three pairs of lung squamous cell carcinoma samples.

File Name: **Supplementary Data 17.**
Description: The TPT value of isoforms in two pairs of OS samples.

File Name: **Supplementary Data 18.**
Description: The DIU genes of two OS samples. C1: OS-1-C; C2: OS-2-C; N1: OS-1-N; N2: OS-2-N. P values were calculated using two-sided Fisher's exact test.

File Name: **Supplementary Data 19**.
Description: The DIU genes of LGC-133 sample. P values were calculated using two-sided Fisher's exact test.

File Name: **Supplementary Data 20**.
Description: The DIU genes of LGC-265 sample. P values were calculated using two-sided Fisher's exact test.

File Name: **Supplementary Data 21**.
Description: The DIU genes of LGC-415 sample. P values were calculated using two-sided Fisher's exact test.

File Name: **Supplementary Data 22**.
Description: The results of RT-qPCR for ECM1b (ECM1-201) and ECM1a (ECM1-202).

File Name: **Supplementary Data 23**.
Description: Cell proliferation assay of cell line 6-10B after transfection in two days.
