# Supplementary material for: TAGET: a toolkit for analyzing full-length transcripts from long-read sequencing
Source: Nat Commun. 2023 Sep 23;14:5935. doi: 10.1038/s41467-023-41649-0 (PMC10518008; doi:10.1038/s41467-023-41649-0)
Supplement: Supplementary file 27 — Reporting Summary [file 41467_2023_41649_MOESM27_ESM.pdf]

nature portfolio

Corresponding author(s): Ruibin Xi

Last updated by author(s): Sep 6, 2023

Reporting Summary

Nature Portfolio wishes to improve the reproducibility of the work that we publish. This form provides structure for consistency and transparency in reporting. For further information on Nature Portfolio policies, see our Editorial Policies and the Editorial Policy Checklist.

Statistics

For all statistical analyses, confirm that the following items are present in the figure legend, table legend, main text, or Methods section.

n/a	Confirmed	
☐	☒	The exact sample size (*n*) for each experimental group/condition, given as a discrete number and unit of measurement
☐	☒	A statement on whether measurements were taken from distinct samples or whether the same sample was measured repeatedly
☐	☒	The statistical test(s) used AND whether they are one- or two-sided<br>*Only common tests should be described solely by name; describe more complex techniques in the Methods section.*
☒	☐	A description of all covariates tested
☐	☒	A description of any assumptions or corrections, such as tests of normality and adjustment for multiple comparisons
☐	☒	A full description of the statistical parameters including central tendency (e.g. means) or other basic estimates (e.g. regression coefficient) AND variation (e.g. standard deviation) or associated estimates of uncertainty (e.g. confidence intervals)
☐	☒	For null hypothesis testing, the test statistic (e.g. *F*, *t*, *r*) with confidence intervals, effect sizes, degrees of freedom and *P* value noted<br>*Give P values as exact values whenever suitable.*
☒	☐	For Bayesian analysis, information on the choice of priors and Markov chain Monte Carlo settings
☒	☐	For hierarchical and complex designs, identification of the appropriate level for tests and full reporting of outcomes
☐	☒	Estimates of effect sizes (e.g. Cohen's *d*, Pearson's *r*), indicating how they were calculated

Our web collection on statistics for biologists contains articles on many of the points above.

Software and code

Policy information about availability of computer code

Data collection	no software was used.
Data analysis	TAGET can be downloaded from https://github.com/XiDsLab/TAGET.<br>for other software: Minimap2 v2.24; HISAT2 v2.2.1; SQANTI2 v7.4.0; DEGseq v3.15; JAFFAL v2.3; GMAP v2017-11-15.

For manuscripts utilizing custom algorithms or software that are central to the research but not yet described in published literature, software must be made available to editors and reviewers. We strongly encourage code deposition in a community repository (e.g. GitHub). See the Nature Portfolio guidelines for submitting code & software for further information.

Data

Policy information about availability of data

All manuscripts must include a data availability statement. This statement should provide the following information, where applicable:

- Accession codes, unique identifiers, or web links for publicly available datasets
- A description of any restrictions on data availability
- For clinical datasets or third party data, please ensure that the statement adheres to our policy

Datasets of COLO829 from the PacBio platform used in this study are available at https://www.zenodo.org/record/8319497.
COLO829 101 bp Illumina short reads are available in NCBI database with an accession number SRR8615617 (https://www.ncbi.nlm.nih.gov/sra/?term=SRR8615617%20). PacBio long-read sequencing and RNA-seq from laryngeal cancer patients, osteosarcoma, and lung squamous cell carcinoma patients

generated in this study have been deposited in GSA database under accession code HRA002806 (https://ngdc.cncb.ac.cn/gsa-human/browse/HRA002806). GM12878 datasets from the ONT platform are available in Gene Expression Omnibus (GEO) with an accession number GSE132766 (https://www.ncbi.nlm.nih.gov/geo/query/acc.cgi?acc=GSE132766). Lung cancer cell lines from the ONT platform are available in DNA Data Bank of Japan (DDBJ) with an accession number DRA001859 (https://ddbj.nig.ac.jp/resource/sra-submission/DRA001859). The data HRA002806 in GSA are available under restricted access for privacy protection. Access for research purposes can be obtained by completing the application form via GSA. Users can register and login to GSA [https://ngdc.cncb.ac.cn/gsa-human/] and follow the guidance of "Request Data" [https://ngdc.cncb.ac.cn/gsa-human/document/GSA-Human_Request_Guide_for_Users_us.pdf] to request the data. Other data used in this study are publicly available. The authors declare that all data supporting the findings described in this manuscript are available in the article and its Supplementary Information files, and from the corresponding author upon request.

Research involving human participants, their data, or biological material

Policy information about studies with human participants or human data. See also policy information about sex, gender (identity/presentation), and sexual orientation and race, ethnicity and racism.

Reporting on sex and gender	Sex and gender has not been collected.
Reporting on race, ethnicity, or other socially relevant groupings	Race, ethnicity, or other socially relevant groupings have not been collected.
Population characteristics	population characteristics has not been collected.
Recruitment	7 tumor tissues surgically resected from patients with LGC, OS, and LUSC were randomly selected with the criterion that the biopsies passed the quality control for Iso-seq. All patients in this study signed a written informed consent. Because all the participants were recruited from the Harbin area(China), they might not have been representative of the wider population. No other potential self-selection bias was present in this study. We used the Iso-seq data from the patients to benchmark the computational toolkit developed in this study. The potential selection bias is unlikely to influence the benchmarking results.
Ethics oversight	The study was conducted in accordance with the ethical standards of the Research Ethics Committee of Harbin Medical University Cancer Hospital with patient's informed content. (Approval No: JJ2022LH1305)

Note that full information on the approval of the study protocol must also be provided in the manuscript.

Field-specific reporting

Please select the one below that is the best fit for your research. If you are not sure, read the appropriate sections before making your selection.

☒ Life sciences ☐ Behavioural & social sciences ☐ Ecological, evolutionary & environmental sciences

For a reference copy of the document with all sections, see nature.com/documents/nr-reporting-summary-flat.pdf

Life sciences study design

All studies must disclose on these points even when the disclosure is negative.

Sample size	No sample-size calculation was performed. This study is about a computational toolkit for Iso-seq data analysis. The purpose of using different types of tumor samples in this study is to validate that this toolkit can be applied to different tumors. To demonstrate the general applicability to Iso-seq tumor data, we aimed to have at least three different tumor types, and each tumor type have at least one pair of tumor samples. Thus in total we have seven pairs of tumor samples.
Data exclusions	No data were excluded.
Replication	Transwell assays experiememts were performed three times to validate the effect of ECM1-201 and ECM1-202 on 6-10B, HeyA8, AU565, and 5-8F cell migration and invasion.
Randomization	Not relevant. The study does not involve different experimental groups.
Blinding	Not relevant. No group allocation is involved in the study.

Reporting for specific materials, systems and methods

We require information from authors about some types of materials, experimental systems and methods used in many studies. Here, indicate whether each material, system or method listed is relevant to your study. If you are not sure if a list item applies to your research, read the appropriate section before selecting a response.

Materials & experimental systems

n/a	Involved in the study
☒	☐ Antibodies
☐	☒ Eukaryotic cell lines
☒	☐ Palaeontology and archaeology
☒	☐ Animals and other organisms
☒	☐ Clinical data
☒	☐ Dual use research of concern
☒	☐ Plants

Methods

n/a	Involved in the study
☒	☐ ChIP-seq
☒	☐ Flow cytometry
☒	☐ MRI-based neuroimaging

Eukaryotic cell lines

Policy information about cell lines and Sex and Gender in Research

Cell line source(s)	Human cell lines COLO829, 6-10B, HeyA8, AU565, and 5-8F were purchased from the Center for Basic Medical Cells, Peking Union Medical College.
Authentication	Cell line authentication services of 6-10B, HeyA8 and 5-8F were provided by Shanghai Yaji Biotechnology Co.,.Ltd
Mycoplasma contamination	All cell lines tested negative for mycoplasma contamination.
Commonly misidentified lines (See ICLAC register)	No commonly misidentified cell lines were used.
